# Comparison of treatment outcomes between combined chemotherapy-radiation therapy (chemo-RT) and radiation therapy alone (RT) for intracranial germ cell tumors in adolescent and young adult patients (AYA)

**DOI:** 10.1007/s12672-025-02103-3

**Published:** 2025-03-25

**Authors:** Warissara Rongthong, Nan Suntornpong, Kullathorn Thephamongkhol, Teeradon Treechairusame

**Affiliations:** Division of Radiation Oncology, Department of Radiology, Faculty of Medicine Siriraj Hospital, Mahidol University, 2 Wang Lang Road, Siriraj, Bangkok Noi, Bangkok, 10700 Thailand

**Keywords:** Intracranial germ cell tumor, Adolescent and young adult, Chemotherapy, Radiotherapy, Nongerminomatous germ cell

## Abstract

**Background:**

The incidence of intracranial germ cell tumors (iGCTs) in adolescents and young adults (AYA) is lower than that in pediatric patients. However, the recurrence rate of iGCT in AYA patients (7.6%) is higher than in children (2%). The use of iGCTs in the AYA population lacks randomized trials to standardize treatment. Therefore, this study aimed to determine the patterns of practice and outcomes of iGCT in AYA.

**Methods:**

This single-center retrospective cohort study iGCT patients aged 15 to 39 who were treated at Siriraj Hospital, Thailand, from 2007 to 2019. The patients' charts were reviewed, and the results were compared between those who received chemotherapy combined with radiotherapy (Chemo-RT) and those who received RT alone.

**Results:**

The median follow-up time was 7.6 years. Eighty-four patients were included in this study: 60 with germinomas and 24 with nongerminomatous germ cell tumors (NGGCT). In the case of NGGCT, the 10-year event-free survival (EFS) and overall survival (OS) were 100% and 100%, respectively, with RT alone. For Chemo-RT, the 10-year EFS was 54.05%, and the 10-year OS was 68.44% (P = 0.640 for EFS and 0.454 for OS). For germinomas, the 10-year EFS was 76.87% with RT alone, and the 10-year OS was 86.40%. For Chemo-RT, the 10-year EFS was 69.63%, and the 10-year OS was 69.63% (P = 0.335 for EFS and 0.022 for OS). Compared with those in the groups treated with Chemo-RT and RT alone adjusted for age > 18 years, primary site, metastasis, type of surgery, field of radiotherapy, sex, serum beta-HCG, and serum AFP, the hazard ratio (HR) of EFS was 2.49 (0.85–7.29) (P = 0.095) and the OS was 2.55 (P = 0.237).

**Conclusions:**

To the best of our knowledge, we present findings on the outcomes of iGCT patients in the AYA age group. After adjusting the hazard ratio, we found no significant difference between patients who received chemotherapy and those who underwent radiotherapy alone in terms of event-free survival and overall survival. Standardized long-term term survival follow-up and supportive treatment in AYA group is needed to improve the outcome and minimize toxicity in this group. There is a need for further randomized control trials that specifically address the population of patients with AYA to improve our understanding of their potential treatment approaches.

**Supplementary Information:**

The online version contains supplementary material available at 10.1007/s12672-025-02103-3.

## Introduction

An intracranial germ cell tumor (iGCT) is less common in adolescents and young adults (AYA) than in pediatric patients [1] representing 0.5–3% of all primary brain tumors. iGCT is more common in Asian populations than in European and North American populations. The prognosis of diseases is based on the histological subtype, biomolecular markers, and treatment [2, 3]. iGCTs are classified into two major groups: germinomas and nongerminomatous germ cell tumors (NGGCT). In addition, age is an essential factor that can affect patient survival [4, 5]. The recurrence rate of iGCT in AYA patients (7.6%) is also higher than the recurrence rate of pediatric iGCT patients (2%) [2, 3, 6]. The paradigm of pediatric iGCT treatment includes surgery, chemotherapy (CMT), and radiotherapy (RT). In contrast, iGCT in the AYA population, defined as individuals aged 15 to 39 years [7], currently lacks prospective well-controlled trials to standardize treatment. Retrospective studies have shown that the use of radiation treatment alone is an effective approach that leads to significant success rates of 96–100% in curing the condition and safety of germinoma [8–10]. Given the favorable outcomes achieved by radiotherapy alone, the use of chemotherapy is not recommended routinely for patients with AYA diagnosed with intracranial germinoma [11–13]. Some trials have shown that combining chemotherapeutic schemes with focal radiotherapy does not improve relapse-free survival [12, 14, 15]. Unlike the pediatric population, AYA patients can generally tolerate radiotherapy well, but the potential for harmful side effects from chemotherapy is more pronounced [15, 16]. Thus, the primary focus of this study was to compare the outcomes of combined chemotherapy and radiotherapy (Chemo-RT) with those of radiotherapy alone.

## Methods and materials

The study adhered to the principles set by the Declaration of Helsinki and approved by the Siriraj Ethics Review Board (protocol number No. Si 358/2020). It is a retrospective single-center cohort at Siriraj Hospital, Mahidol University, Thailand. Informed consent was waived by the Siriraj Ethics Review Board due to the study’s retrospective review and minimal risk to participants. All patients who were diagnosed with iGCT between 15 and 39 years of age at Siriraj Hospital, Thailand, from January 2007 to December 2019 were included in this study. The charts were retrospectively reviewed, and data were collected on event-free survival and overall survival based on cell type. Patients older than 18 years were treated by adult medical oncologists, whereas those younger than 18 years were treated by pediatric oncologists. The diagnoses were made based on pathologic findings, CT/MR images in the brain and spinal regions, and tumor markers according to the Thai National Protocol for the Treatment of Childhood Cancer 2018 and the Children's Oncology Group (COG) [17] following these criteria.

Germinomas should meet the criteria of a pathologic report confirming normal tumor marker levels (serum and/or cerebrospinal fluid (CSF) alpha-fetoprotein (AFP) ≤ 10 ng/dL, serum and/or CSF beta subunit of human chorionic gonadotrophin (B-HCG) ≤ 100 IU/L). In contrast, NGGCT must have a pathological report confirming an immature teratoma with malignant transformation, yolk sac tumor, embryonal cell carcinoma, choriocarcinoma, and mixed germ cell tumor or serum and/or CSF tumor marker AFP > 10 ng/dL or B-HCG > 100 IU/L. Baseline investigations, including serum blood chemistry, complete blood count, serum AFP, serum B-HCG, brain and spine MRI, ophthalmologic evaluation, pituitary axis function evaluation, and biopsy, if indicated, were performed for patients with metastasis at the time of diagnosis.

### Treatment

The treatment sequence and protocols of iGCT have varied based on evolving evidence. At our institution, patients are treated by adult and pediatric oncologists, depending on their age. Thus, various treatment approaches can be used. Patients were treated with various chemotherapy regimens, including etoposide and cisplatin with or without bleomycin (BEP) or carboplatin and etoposide with or without ifosfamide (ICE). The following factors, including elevated tumor markers, the NGCCT subtype, and metastasis, are considered for Chemo-RT treatment. Different chemotherapy protocols consisted of SIOP CNS CGT 96 [14] ICE: carboplatin (600 mg/m2/day, Days 1–3), etoposide (100 mg/m2/day, Days 1–3), ifosfamide (1,800 mg/m2/day, Days 1–5), ACNS 1123 protocol[17] A: etoposide (150 mg/m2/day, Days 1–3) and carboplatin (600 mg/m2/day, Day 1). Cycle B: Etoposide (100 mg/m2/day, Days 1–5), ifosfamide (1,800 mg/m2/day, Days 1–5), and UK CCSG [18, 19] were adopted from extracranial germ cell tumors (cisplatin (100 mg/m2, Day 1), etoposide (120 mg/m2/day, Days 1–3) and bleomycin (15 mg/m2, Day 2)). After four chemotherapy courses, patients underwent serum tumor marker testing and imaging to evaluate the response.

Radiotherapy was delivered at 1.8–2 Gy per fraction. Patients diagnosed with germinoma received whole-ventricular radiotherapy (18–30 Gy)[20] with or without focal boost. Patients diagnosed with NGGCT could undergo 30–36 Gy craniospinal irradiation with or without focal boost, whole-brain irradiation, or only focal boost, depending on postchemotherapy tumor responses and clinician decisions [3, 17, 20]. A focal boost was performed to a cumulative dose of 24–36 Gy in germinoma patients and 45–54 Gy in NGGCT patients.

After treatment was complete, patients were observed with a clinical examination of serum tumor markers at 3- to 4-month intervals for 2 years and then every 6-month interval thereafter. Brain magnetic resonance imaging (MRI) and whole spine screening were performed at 6-month intervals for 2 years and then annually thereafter. Additional MRI findings were investigated depending on the patient's symptoms suspicious of recurrence. Between 2007 and 2019, our institution did not routinely conduct follow-up survivorship screenings for late complications, including neurological impairment, endocrine and growth assessments. Beginning in 2019, we implemented screening for late complications following radiotherapy treatment in pediatric and AYA patients.

### Study design

The primary outcome of the study was overall survival, which was defined as the time interval from the date of completion of radiotherapy to the date of death from any cause; event-free survival, which was defined as the time interval from the date of completion of radiotherapy to the date of recurrence; and death events, which were divided into three subgroups: in-field recurrence, out-field recurrence, and systemic recurrence. In-field recurrence was determined as a site of recurrence within 80% of the prescription isodose line, whereas out-field recurrence was explained as a site of recurrence outside 80% of the prescription isodose line. Systemic recurrence was defined as any recurrence away from the brain or the cerebrospinal system.

### Statistical analysis

The study results were calculated using STATA 16.0. The baseline patient, tumor, and treatment characteristics of the patients were reported using descriptive statistics. The final analysis of overall survival and event-free survival was performed using the Kaplan‒Meier method and log-rank tests to compare the study groups. Univariable and multivariable Cox regression analyses were compared between the two groups. The results were considered significant if P was ≤ 0.05.

## Results

Patient characteristics are shown in Table 1. The median interval from the beginning of treatment to the last follow-up was 7.6 years. A total of 84 patients were enrolled in this study, consisting of 24 diagnosed with NGGCT and 60 with germinoma. A total of 91.6% of patients were diagnosed by pathology, whereas 8.3% were diagnosed based on imaging and tumor markers. The median age of patients is 16 years (15–21), with 79% male (N = 66) and 21% female. In the germinoma group, 24 out of 60 (40%) were older than 18 years (10 in the chemotherapy group; and 14 in the no chemotherapy group, respectively). In the NGGCT group, 7 out of 24 (29%) were older than 18 years (2 in the chemotherapy group; and 5 in the chemotherapy group, respectively). The most common primary site was the pineal region (52%), followed by the suprasellar region (29%).

**Table 1:** Baseline patient status and tumor characteristics

Diagnosis		NGGCT (N = 24)		P-value	Pure germinoma (N = 60)		P-value
		No CMT (N = 4)	CMT (N = 20)		No CMT (N = 24)	CMT (N = 36)	
Age at treatment, year, Median (IQR)		21.5 (16.5–26.5)	15.5 (15–18.5)	0.18	16 (15–23)	17.5 (15–21)	0.96
Sex	Male	4 (100%)	15(75%)	0.54	20 (83%)	27 (75%)	0.53
	Female	0 (0%)	5 (25%)		4 (17%)	9 (25%)	
Pathologic diagnosis	No	0 (0%)	0 (0%)		0 (0%)	7 (19%)	0.035*
	Yes	4 (100%)	20(100%)		24 (100%)	29 (81%)	
Type of surgery	Biopsy	2 (50%)	9 (45%)	1.00	20 (83%)	27 (93%)	0.39
	STR	2 (50%)	11 (55%)		4 (17%)	2 (7%)	
Weight (Kg), Mean (SD)		66.1 (16.7)	47.6 (30.9)	0.33	65.8 (14.3)	55.1 (28.1)	0.13
Height (Cm), Mean (SD)		171 (7.7)	137.4 (30.9)	0.05	170.5 (10.5)	151.6 (26.1)	0.004
Serum AFB, Median (IQR)		2.89 (1.325–17.94)	43.9 (16.405–332.05)	0.04	1.61 (0.8–2.33)	1.395 (0.78–3.22)	0.98
Serum Beta-HCG, Median (IQR)		0.1 (.095–243.5)	53.475 (12.37–750.5)	0.07	1.58 (0.19–26.45)	2.68 (.405–13.54)	0.80
CSF AFP, Median (IQR)		–	2.34 (.5–14.22)		0.6 (0.5–0.76)	0.5 (0.5–0.6)	0.73
CSF Beta-HCG, Median (IQR)		–	35.96 (29.64–103.4)		12.26 (1.5–32.01)	52.5 (3.39–102.9)	0.20
Metastasis	No	4 (100%)	17 (85%)	1.00	23 (96%)	34 (94%)	1.00
	Yes	0 (0%)	3 (15%)		1 (4%)	2 (6%)	
RT	No	0 (0%)	2 (10%)	1.00	0 (0%)	1 (3%)	1.00
	Yes	4 (100%)	18 (90%)		24 (100%)	35 (97%)	
Site of RT	CSI	0 (0%)	11 (61%)	0.081	5 (21%)	10 (29%)	0.20
	WVRT	3 (75%)	5 (28%)		15 (63%)	23 (68%)	
	WBRT	1 (25%)	1 (6%)		4 (17%)	1 (3%)	
	Focal boost	0 (0%)	1 (6%)		0 (0%)	0 (0%)	
Primary tumor location	Pineal	3 (75%)	10 (50%)	1.00	14 (58%)	17 (47%)	0.20
	Suprasellar	1 (25%)	7 (35%)		8 (33%)	8 (22%)	
	Bifocal	0 (0%)	0 (0%)		0 (0%)	1 (3%)	
	Other	0 (0%)	3 (15%)		2 (8%)	10 (28%)	

Values are expressed as mean ± standard deviation and median (IQR)

Abbreviations: AFP = alpha fetoprotein; Beta-HCG = Beta- human chorionic gonadotropin; CSF = cerebrospinal fluid; CMT = chemotherapy; RT = Radiotherapy; NGGCT = non-germinomatous germ cell tumor

### NGGCT

All patients in this group were diagnosed using tissue pathology and elevated tumor marker levels. Eleven out of 24 patients (46%) underwent biopsy, whereas 13 out of 24 patients (54%) underwent subtotal tumor removal (STR). The median B-HCG level in NGGCT was 37.56 IU/L (0.56–583.20) in the serum and 35.63 IU/L (29.64–103.4) in the CSF. For AFP, the median value was 35.05 ng/ml (3.82–198.38) in the serum and 2.34 ng/ml (0.5–14.22) in the CSF. Metastatic disease was found in 3 of 24 patients (13%) by MR imaging and/or CSF cytology. Patients with metastases received chemotherapy followed by CSI to 36 Gy. Twenty of the 24 patients with NGGCT tumors received chemotherapy (83%), including 25% with BEP, 42% with ICE, and 17% with carboplatin combined with etoposide, and twenty-two of the 24 patients received RT (consisting of 55% CSI, 10% WBRT, 40% WVRT, and 5% involved field) (supplementary 1)***.*** Two patients did not receive radiotherapy because one patient was lost to follow-up, and another patient developed lung fibrosis after receiving BEP for 4 cycles and passed away before starting radiation treatment. Two patients experienced an interruption in their RT regimen, each receiving a radiation dose of less than 18 Gy. Both patients died from pneumonia before they could complete the course of radiation therapy.

**Fig. 1:**
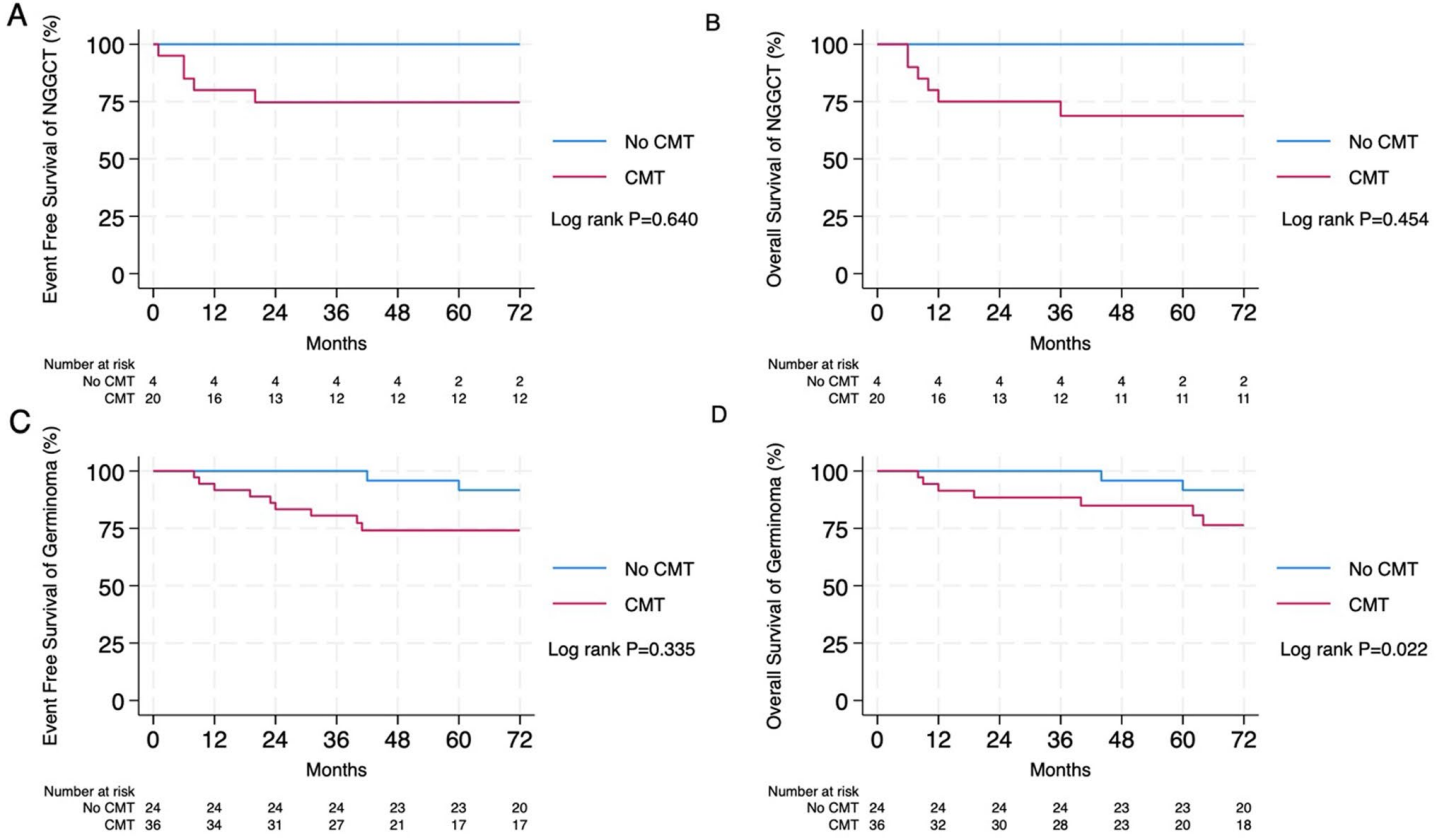
Event-free survival (**1A**) and overall survival (**1B**) of patients with intracranial non-germinomatous germ cell tumor (NGGCT) according to treatment approach, while event-free survival (**1C**) and overall survival (**1D**) of patients with intracranial germinoma tumor (NGGCT) according to treatment approach. Abbreviations: CMT = Chemotherapy; NGGCT = Non-germinomatous germ cell tumor

In NGGCT patients, the 5- and 10-year EFS rates were 73.33% ± 9.3% and 61.11% ± 11.1%, respectively. The 5-year and 10-year OS rates were 75% ± 10.8% and 67.5% ± 12.1%, respectively. For RT alone (N = 4; WVRT 3, WBRT 1), the 5-year and 10-year EFS and OS rates were 100% and 100%, respectively. In the Chemo-RT arm, the 5- and 10-year EFS rates were 67.57% ± 10.88% and 54.05% ± 12.20%, respectively. The 5- and 10-year OS rates were 68.44% ± 10.79% and 68.44% ± 10.79%, respectively with P = 0.640 (Fig. 1A). Three of the 8 deaths were related to disease recurrence. One patient who had a multifocal mixed germ cell tumor combined with germinoma and immature teratoma received CMT with a stable response and passed away due to disease recurrence. The second patient subsequently received CSI treatment for 9 sessions and subsequently passed away from severe pneumonia. The third patient, who had a mixed germ cell tumor with spinal metastasis and paraplegia at presentation, received BEP with CSI to 36 Gy and passed away after 5 months of completing a radiation therapy course due to urinary tract infection with sepsis. Four patients of the 8 deaths experienced severe sepsis after finishing treatment at least one year. Two of them had received BEP and suffered from panhypopituitarism after treatment. One patient developed lung fibrosis and sepsis later. The other patient had a history of severe pneumonia. No documentation about the cause of death was available for one patient (supplementary 2).

**Fig. 2:**
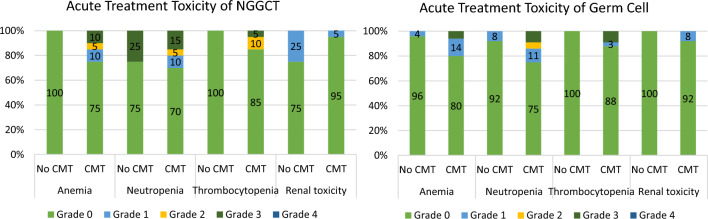
demonstrated the acute toxicities of non-germinomatous germ cell tumor (NGGCT) and germinoma patients between group of received treatment with chemotherapy and without chemotherapy

The five- and 10-year OS rates were 100% and 100%, respectively, for WBRT and WVRT alone; 100% and 75%, respectively, for CSI plus CMT; and 66.67% and 66.67%, respectively, for WVRT + CMT. When we compared the OS of CMT patients with that of RT patients, there were no statistically significant differences in OS (P = 0.640), as shown in Fig. 1B**.** Three patients experienced relapse after completing treatment. All had received combined CMT and radiation. The pattern of failure was in-field RT in 2 of the three patients, following CSI 36Gy with a tumor bed boost to 54Gy, and WVRT with a tumor bed boost to 54Gy. One of the three patients developed diffuse leptomeningeal involvement and local recurrence in the pineal area, as shown in Table 2***.*** Acute toxicities are reported in Fig. 2**.** Grade 3 toxicity was found in patients who received CMT plus RT more than in those who received RT alone in terms of anemia (10%) and thrombocytopenia (5%). The rate of interruption of radiotherapy treatment in the combined Chemo-RT group was 25% (5/20), and that in the RT alone group was 0% (0/4).

**Table 2:** Patients with NGGCT and germinoma who experienced relapse

Age	Primary site	Pretreatment biopsy	Serum B-HCG (IU/L)	Serum AFP (ng/ml)	Baseline spinal imaging	Initial CMT	Initial surgery	Initial RT	Site and timing of initial relapse	Salvage treatment	Outcome
NGGCT											
15	Basal ganglion	Yes	821.5	770.7	Yes	ICE × 5 cycles (PR)	Biopsy	CSI 36 Gy with tumor bed boost to 54 Gy	Basal ganglion at 79 months	Carboplatin plus Paclitaxel 5 cycles followed by Focal RT 36 Gy/12Fr	DOD due to 2nd Relapse at T10 and thecal sac S/P Gemcitabine plus carboplatin and focal RT then developed UTI with severe sepsis 129 months
29	Suprasellar	Yes	27.48	1266	Yes	ICE X 4 cycles (CR)	Biopsy	WVRT 36 Gy	Drop metastases at T5-6 and L1 at 6 months	Laminectomy with tumor removal followed by CSI 36 Gy and boost to 45 Gy	DOD at 35 months
25	Pineal	Yes	9586	19.5	Yes	BEP X 4 cycles (PR)	STR	WVRT 36 Gy with tumor bed boost to 54 Gy	Pineal with diffuse leptomeningeal involvement at 20 months	Carboplatin plus paclitaxel 6 cycles	DOD at 31 months
Germinoma											
22	Pineal	Yes	58.85	5.06	Yes	No	STR	WVRT 30 Gy + Tumor bed boost to 50 Gy	Cevicomedullary junction (out-of-field) at 42 months	Cisplatin plus etoposide 1 cycle, then patient loss follow-up and third relapse with drop metastasis at T1-2 level and thecal sac S/P surgery and RT	Alive without disease at a follow-up time of 194 months
19	Pineal	Yes	0.63	0.68	Yes	BEP × 4 cycles (CR)	Biopsy	WVRT 24 Gy + Tumor bed boost to 46 Gy	L3 drop metastasis at 24 months (out-of-field)	Ifosfamide, cisplatin and vinblastin followd by CIS 23.4 Gy and focal boost to 50 Gy	DOD at 129 months
27	Pineal	Yes	1.21	4.03	Yes	No	Biopsy	WVRT 25.2 Gy + Tumor bed boost to 45 Gy	Rt cerebellum and L1-S2 level At 31 months	BEP 4 cycles then CSI 24 Gy with foacal boost to 36 Gy	Alive with disease recurrent along lateral ventricle and follow up time 135 months
17	Pituitary	Yes	1.94	0.68	Yes	Cisplatin + Etoposide × 4 cycles (CR)	Biopsy	WVRT 24 Gy + Tumor bed boost to 50 Gy	L1 level at 23 months (out-of-field)	ICE 3 cycles followed by CSI 24 Gy with focal boost to 50 Gy	2nd and 3rd relapse at both brain and CSI area then DOD 72 months
16	Periventricular	Yes	58.85	5.06	Yes	Carboplatin + Etoposide × 4 cycles (PD)	Biopsy	WVRT 36 Gy + Tumor bed boost to 50 Gy	Basal ganglion at 41 months	Carboplatin plus Etoposide 4 cycles then plan RT, patient loss follow-up and came bak again 4 month later due to headache then received Carboplatin + Paclitaxel × 6 cycles	DOD at 69 months

Abbreviations: AFP = alpha fetoprotein; Beta-HCG = beta-human chorionic gonadotropin; CMT = chemotherapy; ICE = ifosphamide + carboplatin + etoposide; BEP = bleomycin + etoposide + cisplatin; CSI = craniospinal irradiation; WVRT = whole-ventricle radiotherapy; STR = subtotal resection; DOD = dead of disease; CR = complete response; PR = partial response; PD = progressive disease

For patients in the subgroup with elevated tumor markers, sixteen patients received CMT + RT. The 5-year and 10-year EFS rates were 66.67% ± 13.93% and 66.67% ± 13.93%, respectively. The 5- and 10-year OS rates were 71.38% ± 12.18% and 71.38% ± 12.18%, respectively. Only one patient received radiotherapy alone because of poor performance status. He underwent WBRT 34 Gy in 17 fractions alone. Despite these challenging circumstances, the patient survived for 13 years after completing the radiotherapy course. He passed away from severe sepsis at the primary hospital.

### Germinoma

Fifty-three out of 60 patients underwent surgery, whereas 7 out of 60 patients were diagnosed by radiological findings and tumor marker levels. Forty-seven of the 60 patients had biopsies, and six had subtotal resections (STRs). The median B-HCG concentration was 1.91 IU/L (0.20–17.6) in the serum and 22.46 IU/L (2.90–81.70) in the CSF. For AFP, the median value was 1.47 ng/ml (0.79–2.59) in the serum and 0.5 ng/ml (0.5–0.7) in the CSF. Thirty-six patients (60%) received chemotherapy composed of BEP (25%), carboplatin plus etoposide (14%), cisplatin plus etoposide (31%), or ICE (30%). Fifty-nine patients (97%) received radiotherapy treatment (65% WVRT, 25% CSI, and 8% WBRT) (supplementary 3,4). One patient did not proceed with radiotherapy because the condition continued to worsen after completing four cycles of BEP treatment due to septic shock. This patient subsequently died from obstructive hydrocephalus caused by disease progression in the left basal ganglion, which resulted from delayed treatment. Three of the 60 patients had radiographic spinal metastases and positive CSF cytology. All three patients with metastases received combined CSI and chemotherapy.

In germinoma patients, the EFS rates were 82.7% ± 5% at 5 years and 72% ± 6.7% at 10 years. The 5-year and 10-year OS rates were 89.30% ± 4.1% and 77.19% ± 6.23%, respectively. For RT alone, the 5-year and 10-year EFS rates were 95.83% ± 4.08% and 76.87% ± 9.16%, respectively. The 5-year and 10-year OS rates were 95.83% ± 4.08% and 86.40% ± 7.35%, respectively. In contrast, for Chemo-RT, the 5-year and 10-year EFS rates were 73.33% ± 7.69% and 69.63% ± 9.54%, respectively. The 5-year and 10-year OS rates were 85.10% ± 6.19% and 69.63% ± 9.54%, respectively (Fig. 1C and 1D). Of the 15 deaths (range 9–156 months), 6 were related to disease, 1 was related to complications of treatment, 4 were due to severe infection, and 4 were due to unrelated causes (2 from heart disease, 1 from meningitis, and 1 from obstructive sleep apnea -related sequence). Nine patients (60%) had panhypopituitarism with long-term steroid supplementation. The five- and 10-year OS rates were 100% and 100%, respectively, for WBRT plus CMT and CSI alone; 87.5% and 87.5%, respectively, for CSI plus CMT; 93% and 87%, respectively, for WVRT alone; 100% and 75%, respectively, for WBRT alone; and 85% and 62%, respectively, for WVRT + CMT (Table 2). The local pattern of failure was in-field RT in 3 out of 5 patients. In terms of the overall survival of patients with pure germinoma based on the RT site and CMT, no statistically significant differences in OS parameters were observed (P = 0.41). Regarding EFS and OS, the RRs of patients who received combined CMT and RT compared with RT alone were 1.61 (P = 0.308) and 2.74 (P = 0.141), respectively, as shown in Table 3*.* After adjusting for age > 18 years, primary site, metastasis, type of surgery, field of radiotherapy, sex, serum beta-HCG, and serum AFP, the HRs for EFS and OS were 2.49 (P = 0.095) and 2.55 (P = 0.237), respectively.

**Table 3:** Hazard ratio of the effect of chemotherapy on EFS and survival in patients with pure germ cell tumors

Germ cell tumor	HR for EFS survival with 95% CI	P value	HR for Overall survival with 95% CI	P value
RT alone	Baseline		Baseline	
Non-adjusted HR of combined CMT + RT	1.61 (0.64–4.08)	0.308	2.74 (0.72–10.46)	0.141
Adjusted HR				
by age > 18	1.81 (0.69–4.68)	0.221	3.42 (0.87–13.41)	0.078
by age > 18, primary site	1.61 (0.61–4.28)	0.340	2.73 (0.64–11.62)	0.173
by age > 18, primary site, metastasis	1.95 (0.69–5.50)	0.206	2.88 (0.67–12.27)	0.152
by age > 18, primary site, metastasis, type of surgery	2.59 (0.85–7.84)	0.091	3.57 (0.79–16.04)	0.096
by age > 18, primary site, metastasis, type of surgery, field of radiotherapy	2.57 (0.91–7.24)	0.073	2.96 (0.69–12.61)	0.140
by age > 18, primary site, metastasis, type of surgery, field of radiotherapy, sex	2.74 (0.85–7.91)	0.061	3.00 (0.71–12.72)	0.134
by age > 18, primary site, metastasis, type of surgery, field of radiotherapy, sex, serum beta-HCG	2.74 (0.95–7.88)	0.061	3.20	0.125
by age > 18, primary site, metastasis, type of surgery, field of radiotherapy, sex, serum beta-HCG, serum AFP	2.49 (0.85–7.29)	0.095	2.55	0.237

The acute toxicity was lower than that in the nongerminoma group. The rates of grade 2 and 3 toxicities in terms of anemia, neutropenia, thrombocytopenia, and renal toxicity were higher in patients who received RT and CMT than in those who received RT alone, as depicted in Fig. 2***.*** Radiotherapy interruption during Chemo-RT was found in 17.6% (6/34) and 21.7% (5/23) of the patients who received radiotherapy alone.

## Discussion

Our study is a retrospective report on the results of specific iGCTs in the AYA group, which currently has less evidence than the pediatric group to support treatment management [21]. At our institution, patients are treated by either adult or pediatric oncologists, depending on their age. As a result, various treatment approaches can be employed, which can impact outcomes[22]. Improving knowledge about the general outcomes of patients with AYA based on various treatment approaches is essential for standardizing treatment in the AYA group. Wang et al. [5]. showed that in adult patients, RT alone is the most commonly used method. In the pediatric population, the CMT plus RT approach is predominant. The addition of CMT to RT in germinoma can allow a reduction of the dose and volume of irradiation[23]. In the NGGCT group, 20/24 (83%) patients received chemotherapy treatment due to poorer outcomes compared to the germinoma group. However, some studies[5, 24] have indicated no significant disparity in survival rates between the groups that received only RT and those who underwent combined treatment corresponding with our cohort. We reported that the adjusted hazard ratios of the effects of chemotherapy on EFS and OS by age > 18 years, primary site, metastasis, type of surgery, field of radiotherapy, sex, serum beta-HCG, and serum AFP were not significant factors that affected either EFS (hazard ratio 12.49 (0.85–7.29), P = 0.095) or OS (hazard ratio 2.55, P = 0.237). At our institution, compared with other studies in developed countries[15, 25], the outcomes of iGCTs in AYA are inferior in terms of EFS and OS, especially in the NGGCT subgroup and over 10 years. A previous study in Thailand focused on pediatric populations under 15 years of age [26] reported 5-year EFS and OS rates of 94.3% and 96.2%, respectively, which were higher than those reported in our study in AYA groups. A study by Pramesh CS et al.[27] reported that survival rates in low-middle-income countries are lower than those in high-income countries. The factor that could affect survival outcomes is delayed diagnosis from the primary hospital to the tertiary hospital. The delay in diagnosis of an intracranial germ cell tumor can potentially increase the risk of disseminated disease [28]. Our results revealed that 9 of 14 patients (64%) died from severe infection, corresponding to a 10-year OS decrease compared with that reported in another study [15, 29]. B S Ehrlich et al. [30] reported that treatment-related mortality was inversely related to country income. The most common cause of death was sepsis (71.7%). One important factor that should be considered is hypopituitarism, which can be found in pediatric brain tumors and can cause increased mortality[31, 32]. In our cohort, we should standardize our cancer follow-up schedule including endocrinological, and infection prevention to improve survival outcomes and minimize treatment-related mortality in the AYA population. Higher RT doses are often associated with more long-term complications which poses problems for independent living even more so problematic in a middle-income country. Furthermore, the interruption of radiotherapy is a factor that affects the outcome of local control and overall survival [33].

The standard treatment strategy for intracranial germ cell tumor patients involves the integration of chemotherapy and radiation, supported by a substantial body of randomized evidence. However, radiation therapy treatment strategies also differ between the SIOP and COG approaches [14]. For NGGCT, following the SIOP protocol, the treatment plan employs a radiation delivery plan to the involved field RT up to a dose of 54 Gy [3, 14, 34]. In contrast, the COG method, as described in the ACNS0122 protocol, begins 6 cycles of CMT followed by CSI at a dose of 36 Gy, followed by a subsequent dose boost of up to 54 Gy due to the increasing number of patients who experienced a relapse in the spinal area compared with historical data [17]. In ACNS1123, patients who achieved a complete response (CR) or partial response (PR) after six cycles of CMT with carboplatin and etoposide using reduced-dose radiation, defined as 30.6 Gy to WVRT and a 54 Gy boost in the tumor bed, were compared with those in the ACNS0122 cohort. Comparable progression-free survival rates were reported, but the pattern of failure in ACNS 1123 patients was distant spinal relapse at 9% [17]. Because the distant spinal relapse rate after WVRT is still high, ACNS2021 will invest in ventricular plus spinal canal irradiation and pattern failure after induction CMT with a CR or PR. For pure germinoma, for SIOP-CNS-GCT 96 [14], the treatment involves delivering a 24-Gy WVRT dose, followed by a focused boost of RT to 40 Gy if a CR is not achieved. On the other hand, COG follows a WVRT dose of 18 Gy, along with a focal RT boost to a total of 30–36 Gy after CR is achieved. If no complete response is achieved, the COG approach adjusts the treatment to a dose of WVRT of 24 Gy, followed by a boost of 36 Gy [35, 36]. In contrast, the AYA population faces a scarcity of well-established approaches due to a lack of robust evidence, so radiotherapy approaches at our institution vary across individual patients. Although the SIOP-CNS-GCT 96 protocol has no age limit, most patients in the study were between 16 and 23 years of age, with only one patient aged 30 years. The adoption of a protocol for AYA (adolescent and young adult) individuals remains a concern for practical application [3]. Patients with NGGCT who are treated with focal tumor bed and WBRT appear to have a shorter OS than those treated with WVRT and CSI. Only one patient developed an out-of-field relapse after WVRT. This finding might support the findings of Fonseca et al.[37], who reported that relapse patterns are not associated with the field of radiation therapy. However, this difference was not statistically significant (P = 0.76), possibly due to the limited size of the sample used for the analysis. More studies should focus on this topic. Considering the pros and cons between RT alone vs. chemotherapy and RT, it is important to advocate for appropriate management in AYA patients.

## Conclusions

This study could help bridge the gap in practice patterns and outcomes of the AYA iGCT population. Our results showed that germinoma and NGGCT have different responses depending on the multiple factors involved in treatment and patient prognosis. Although there are many differences in the treatment approaches used for iCGT worldwide, the EFS and OS for germinomas seem superior to those for NGGCT. The EFS and OS for both germinoma patients and NGGCT patients treated with radiation alone were similar to those treated with CMT. After adjusting the hazard ratio, we found no significant difference between patients who received chemotherapy and those who underwent radiotherapy alone in terms of event-free survival and overall survival. Owing to the rarity of this disease in the AYA population, conducting specific trials tailored to AYA can be a formidable challenge. It is imperative to conduct more research on this demographic data to gather more comprehensive information that can help solidify the treatment approach for patients with AYA. Standardized long-term term survival follow-up in AYA group is needed to improve the outcome and minimize toxicity in this group.

### Limitations

The limitations of this study include its nonrandomized retrospective design, as well as the possibility of selection bias in treatment approaches. Consequently, several other disease factors could probably lead physicians to intensify aggressive treatment options. Additionally, the sample size is a constraint, especially regarding the limited number of cases involving NGGCTs upon which we base our conclusions.

## Supplementary Information

Additional file1 (DOCX 79 kb)

## Data Availability

Research data are stored in an institutional repository and will be revealed upon request to the corresponding author, Teeradon Treechairusame.
