# Supplementary material for: Comparison of treatment outcomes between combined chemotherapy-radiation therapy (chemo-RT) and radiation therapy alone (RT) for intracranial germ cell tumors in adolescent and young adult patients (AYA)
Source: Discov Oncol. 2025 Mar 25;16:391. doi: 10.1007/s12672-025-02103-3 (PMC11937473; doi:10.1007/s12672-025-02103-3)
Supplement: Supplementary file 1 — Additional file1 (DOCX 79 kb) [file 12672_2025_2103_MOESM1_ESM.docx]

**Supplementary Material**

**Supplementary 1: Treatment of NGGCT, including Surgery, Chemotherapy, and Radiotherapy modalities**

**Supplementary 2: Cause of death of intracranial germ cell tumor**

**Supplementary 3: Treatment of germinoma including Surgery, Chemotherapy, and Radiotherapy modalities**

**Supplementary 4: Diagram showing the treatment approaches of intracranial germ cell tumor at our institution**

**Supplementary 1: Treatment of NGGCT, including Surgery, Chemotherapy, and Radiotherapy modalities**

| Site of RT |  | CSI | WBRT | WVRT | Involved field | No RT | p value |
| --- | --- | --- | --- | --- | --- | --- | --- |
|  |  | N = 11 | N = 2 | N = 8 | N = 1 | N=2 |  |
| Surgery |  | 11 (100%) | 2 (100%) | 8 (100%) | 1 (100%) | 2 (100%) |  |
| Type of surgery | Biopsy | 7 (64%) | 2 (100%) | 2 (29%) | 0 (0%) | 0 (0%) | 0.095 |
|  | STR | 4 (36%) | 0 (0%) | 5 (71%) | 1 (100%) | 2 (100%) |  |
| Chemotherapy | Yes | 11 (100%) | 1 (50%) | 5 (62%) | 1 (100%) | 2 (100%) | 0.14 |
|  | No | 0 (0%) | 1 (50%) | 3 (38%) | 0 (0%) | 0 (0%) |  |
| Chemotherapy regimen | BEP | 2 (18%) | 1 (50%) | 2 (25%) | 0(0%) | 1 (50%) | 0.097 |
|  | Carbo+Eto | 1 (9%) | 0 (0%) | 1 (12%) | 1 (100%) | 1 (50%) |  |
|  | ICE | 8 (73%) | 0 (0%) | 2 (25%) | 0 (0%) | 0 (0%) |  |
| RT Technique | 2D | 1 (9%) | 1 (50%) | 1 (12%) | 0 (0%) | 0 (0%) | 0.49 |
|  | 3D | 6 (55%) | 1 (50%) | 2 (25%) | 0 (0%) | 0 (0%) |  |
|  | IMRT | 4 (36%) | 0 (0%) | 5 (62%) | 1 (100%) | 0 (0%) |  |
| Phase 1 RT dose | < 18 Gy | 2 (18%) | 0 (0%) | 0 (0%) | 0 (0%) | 2 (100%) | 0.12 |
|  | 18-30 Gy | 1 (9%) | 0 (0%) | 1 (12%) | 0 (0%) | 0 (0%) |  |
|  | >30 Gy | 8 (73%) | 2 (100%) | 7 (88%) | 1 (100%) | 0 (0%) |  |
| Tumor bed boost | Yes | 9 (82%) | 1 (50%) | 1 (12%) | 1 (100%) | 2 (100%) | 0.051 |
|  | No | 2 (18%) | 1 (50%) | 7 (88%) | 0 (0%) | 0 (0%) |  |
| Phase 2 RT dose | <50 Gy | 3 (38%) | 0 (0%) | 3 (43%) | 0 (0%) | 0 (0%) | 0.71 |
|  | >50-54 Gy | 5 (63%) | 1 (100%) | 4 (57%) | 0 (0%) | 0 (0%) |  |
| RT interruption | Yes | 4 (36%) | 0 (0%) | 1 (12%) | 0 (0%) | 0 (0%) | 0.47 |
|  | No | 7 (64%) | 2 (100%) | 7 (88%) | 1 (100%) | 0 (0%) |  |

Abbreviations: CSI = craniospinal irradiation; WBRT =whole-brain radiation; WVRT = whole-ventricle radiotherapy; STR = subtotal resection; BEP = bleomycin+ etoposide+ cisplatin; Carbo+Eto = carboplatin+ etoposide; ICE = ifosphamide+ carboplatin+ etoposide; 2D = two-dimensional radiotherapy; 3D= three-dimensional radiotherapy; IMRT = intensity-modulated radiotherapy.

**Supplement 2: Cause of death of intracranial germ cell tumor**

| **Cause of death** | **NCCGCT (N= 8)** | | **Germinoma (N= 15)** | |
| --- | --- | --- | --- | --- |
|  | **No CMT (N= 0)** | **CMT (N= 8)** | **No CMT (N= 6)** | **CMT (N= 9)** |
| **Cancer-related cause** | 0 (0%) | 3 (37.5%) | 2 (13.3%) | 5 (33.3%) |
| **Infection** | 0 (0%) | 4 (50%) | 1 (6.7%) | 3 (20%) |
| **Heart disease** | 0 (0%) | 0 (0%) | 2 (13.3%) | 0 (0%) |
| **Other cause** | 0 (0%) | 0 (0%) | 1 (6.7%) | 0 (0%) |
| **Unknown** | 0 (0%) | 1 (12.5%) | 0 (0%) | 1 (6.7%) |

**Supplementary 3: Treatment of germinoma, including Surgery, Chemotherapy, and Radiotherapy modalities**

| Site of RT |  | CSI | WBRT | WVRT | No RT | p value |
| --- | --- | --- | --- | --- | --- | --- |
|  |  | N = 15 | N = 5 | N = 39 | N =1 |  |
| surgery | Yes | 12 (80%) | 5 (100%) | 35 (90%) | 1 (100%) | 0.49 |
|  | No | 3 (20%) | 0 (0%) | 4 (10%) | 0 (0%) |  |
| Type of surgery | Biopsy | 9 (75%) | 5 (100%) | 32 (91%) | 1 (100%) | 0.31 |
|  | STR | 3 (27%) | 0 (0%) | 3 (9%) | 0 (0%) |  |
| Chemotherapy | Yes | 10 (67%) | 1 (20%) | 24 (62%) | 1 (100%) | 0.23 |
|  | No | 5 (33%) | 4 (80%) | 15 (38%) | 0 (0%) |  |
| Chemotherapy regimen | BEP | 2 (13%) | 0 (0%) | 7 (18%) | 0 (0%) | 0.12 |
|  | Carbo+Eto | 1 (7%) | 0 (0%) | 4 (10%) | 0 (0%) |  |
|  | Cis+Eto | 1 (7%) | 0 (0%) | 10 (26%) | 0 (0%) |  |
|  | ICE | 6 (40%) | 1 (20%) | 3 (8%) | 1 (100%) |  |
| RT Technique | 2D | 4 (27%) | 1 (20%) | 1 (3%) | 0 (0%) | 0.036 |
|  | 3D | 7 (47%) | 4 (80%) | 21 (54%) | 0 (0%) |  |
|  | IMRT | 4 (27%) | 0 (0%) | 17 (44%) | 0 (0%) |  |
| Phase 1 RT dose | 18-23 Gy | 2 (13%) | 0 (0%) | 4 (10%) | 0 (0%) | 0.69 |
|  | $\geq24$ Gy | 13 (87%) | 5 (100%) | 35 (90%) | 0 (0%) |  |
| Tumor bed boost | Yes | 14 (93%) | 5 (100%) | 39 (100%) | 0 (0%) | <0.001 |
|  | No | 1 (7%) | 0 (0%) | 0 (0%) | 0 (0%) |  |
| Phase 2 RT dose | <40 Gy | 1 (7%) | 0 (0%) | 2 (5%) | 0 (0%) | 0.22 |
|  | 40-53 Gy | 6 (43%) | 4 (80%) | 28 (74%) | 0 (0%) |  |
|  | $\geq54$Gy | 7 (50%) | 1 (20%) | 9 (23%) | 0 (0%) |  |
| RT interruption | Yes | 4 (27%) | 0 (0%) | 7 (18%) | 0 (0%) | 0.42 |
|  | No | 11 (73%) | 5 (100%) | 32 (82%) | 0 (0%) |  |

Abbreviations: CSI = craniospinal irradiation; WBRT =whole-brain radiation; WVRT = whole-ventricle radiotherapy; STR = subtotal resection; BEP = bleomycin+ etoposide+ cisplatin; Carbo+Eto = carboplatin+ etoposide; ICE = ifosphamide+ carboplatin+ etoposide; 2D = two-dimensional radiotherapy; 3D= three-dimensional radiotherapy; IMRT = intensity-modulated radiotherapy.

**Supplementary 4: Diagram showing the treatment approaches of intracranial germ cell tumor at our institution**

Enrolled (N = 84)

Germinoma (N = 60)

NGGCT (N = 24)

CR

(N = 4)

PR

(N=5)

SD

(N=8)

PD

(N=3)

CMT

(N=20)

No CMT

(N=4)

WVRT (N=3)

WBRT (N=1)

WVRT (N=3)

CSI (N=1)

CSI (N=2)

WVRT (N=1)

WBRT (N=1)

Focal (N=1)

CSI (N=6)

WVRT (N=1)

No RT (N=1)

CSI (N=2)

No RT (N=1)

CMT

(N=36)

No CMT

(N=24)

WVRT (N=15)

CSI (N=5)

WBRT (N=4)

CR

(N = 11)

PR

(N=15)

SD

(N=5)

PD

(N=2)

WVRT (N=8)

CSI (N=3)

WVRT (N=8)

CSI (N=6)

WBRT (N=1)

WVRT (N=4)

CSI (N=1)

WVRT (N=1)

No RT (N=1)

No imaging response evaluation

(N=3)

WVRT (N=3)
